# Maternal iodine status, intrauterine growth, birth outcomes and congenital anomalies in a UK birth cohort

**DOI:** 10.1186/s12916-020-01602-0

**Published:** 2020-06-11

**Authors:** Charles Jonathan Peter Snart, Diane Erin Threapleton, Claire Keeble, Elizabeth Taylor, Dagmar Waiblinger, Stephen Reid, Nisreen A. Alwan, Dan Mason, Rafaq Azad, Janet Elizabeth Cade, Nigel A. B. Simpson, Sarah Meadows, Amanda McKillion, Gillian Santorelli, Amanda H. Waterman, Michael Zimmermann, Paul M. Stewart, John Wright, Mark Mon-Williams, Darren Charles Greenwood, Laura J. Hardie

**Affiliations:** 1 Leeds Institute of Cardiovascular & Metabolic Medicine, LIGHT Laboratories, School of Medicine, University of Leeds, Leeds, LS2 9JT UK; 2 Leeds Institute for Data Analytics, University of Leeds, Leeds, LS2 9JT UK; 3 Bradford Institute for Health Research, Bradford Teaching Hospitals NHS Foundation Trust, Bradford, BD9 6RJ UK; 4 Earth Surface Science Institute, School of Earth and Environment, University of Leeds, Leeds, LS2 9JT UK; 5 School of Primary Care and Population Sciences, Faculty of Medicine, Southampton General Hospital, University of Southampton, Southampton, SO16 6YD UK; 6 NIHR Southampton Biomedical Research Centre, University of Southampton and University Hospital Southampton NHS Foundation Trust, Southampton, UK; 7 Nutritional Epidemiology Group, School of Food Science & Nutrition, University of Leeds, Leeds, LS2 9JT UK; 8 Division of Women’s and Children’s Health, School of Medicine, University of Leeds, Leeds, LS2 9JT UK; 9 Elsie Widdowson Laboratory, University of Cambridge, Cambridge, CB1 9NL UK; 10 NIHR Nutritional Biomarker Laboratory, MRC Epidemiology Unit, University of Cambridge, Clifford Allbutt Building, Hills Road, Cambridge, CB2 0AH UK; 11 School of Psychology, University of Leeds, Leeds, LS2 9JT UK; 12 Laboratory for Human Nutrition, Institute of Food, Nutrition and Health, ETH Zurich, 8092 Zürich, Switzerland; 13 Faculty of Medicine and Health, University of Leeds, Leeds, LS2 9JT UK

**Keywords:** Birthweight, Iodine, Pregnancy, Insufficiency, SGA

## Abstract

**Background:**

Severe iodine insufficiency in pregnancy has significant consequences, but there is inadequate evidence to indicate what constitutes mild or moderate insufficiency, in terms of observed detrimental effects on pregnancy or birth outcomes. A limited number of studies have examined iodine status and birth outcomes, finding inconsistent evidence for specific outcomes.

**Methods:**

Maternal iodine status was estimated from spot urine samples collected at 26–28 weeks’ gestation from 6971 mothers in the Born in Bradford birth cohort. Associations with outcomes were examined for both urinary iodine concentration (UIC) and iodine-to-creatinine ratio (I:Cr). Outcomes assessed included customised birthweight (primary outcome), birthweight, small for gestational age (SGA), low birthweight, head circumference and APGAR score.

**Results:**

There was a small positive association between I:Cr and birthweight in adjusted analyses. For a typical participant, the predicted birthweight centile at the 25th percentile of I:Cr (59 μg/g) was 2.7 percentage points lower than that at the 75th percentile of I:Cr (121 μg/g) (99% confidence interval (CI) 0.8 to 4.6), birthweight was predicted to be 41 g lower (99% CI 13 to 69) and the predicted probability of SGA was 1.9 percentage points higher (99% CI 0.0 to 3.7). There was no evidence of associations using UIC or other birth outcomes, including stillbirth, preterm birth, ultrasound growth measures or congenital anomalies.

**Conclusion:**

Lower maternal iodine status was associated with lower birthweight and greater probability of SGA. Whilst small, the effect size for lower iodine on birthweight is comparable to environmental tobacco smoke exposure. Iodine insufficiency is avoidable, and strategies to avoid deficiency in women of reproductive age should be considered.

**Trial registration:**

ClinicalTrials.gov NCT03552341. Registered on June 11, 2018.

## Background

Iodine is essential for normal thyroid function and demands for iodine increase during pregnancy to compensate for increased renal clearance and to support normal foetal growth and development [1, 2]. The consequences of severe maternal iodine deficiency have long been known and include goitre, stillbirth and severe neurological and growth impairment in the offspring [1, 3]. However, less is known about the more subtle consequences of mild or moderate deficiency during pregnancy. The World Health Organization (WHO) identifies insufficient iodine intake in pregnant populations where the median urinary iodine concentration (UIC) is less than 150 μg/L [3]. However, this threshold is based on theoretical assumptions about absorption, metabolic needs and excretion in urine [1] rather than observed birth or pregnancy outcomes and is therefore limited in application to pregnant populations.

To date, nine studies have examined maternal iodine status and birth outcomes, with inconsistent findings reported between studies and for specific outcomes [4–12]. Three observational studies report that lower UIC is associated with lower birthweight [4, 5, 7], higher risk of small for gestational age (SGA) [4], shorter length at birth [5], smaller head circumference [5] and increased risk of preterm birth [7]. Lower birthweight is an established risk factor for adult chronic disease [13]. However, the remaining six studies did not report evidence of associations for major birth or pregnancy outcomes [6, 8–12].

In countries without routine salt iodisation or fortification programmes, there is a growing concern about possible endemic iodine insufficiency amongst women of reproductive age [1, 14]. For example, of the 31 European countries that assessed iodine intake in pregnancy, two thirds reported inadequate intakes based on urinary iodine excretion (< 150 μg/L) [14]. The current study, commissioned by the Department of Health for England, directly addresses the uncertainty surrounding the potential negative consequences of low maternal iodine status for birth and pregnancy outcomes. This research question is pertinent given that there is no salt iodisation programme or pregnancy-specific recommendations for iodine intake in the UK. We therefore hypothesise that lower maternal iodine status is associated with smaller foetal or birth size and poorer pregnancy outcomes.

## Methods

### Study design and participants

Women were recruited into the Born in Bradford (BiB) cohort (*n* = 12,453) from 2007 to 2010 at 26 to 28 weeks’ gestation. The BiB cohort has been described in detail elsewhere [15]. In brief, the cohort is multi-ethnic with 43% of participants in this study being of White European background and 43% being of Pakistani ethnic background. A total of 7066 urine samples were provided by 6979 mothers (some women participated during successive pregnancies), from which iodine status was calculated. The large study size provides sufficient power for detecting associations even of a potentially modest size (see protocol at ClinicalTrials.gov NCT03552341).

### Assessment of urinary iodine and creatinine

Urine samples were collected at routine antenatal clinics for oral glucose tolerance tests at 26 to 28 weeks’ gestation and therefore were all collected in the morning after overnight fasting. Samples were stored at − 80 °C until iodine and creatinine concentration analysis.

Urinary iodine^(127)^ concentration (μg/L) was measured at the University of Leeds using inductively coupled plasma mass spectrometry (Thermo iCAP Q, Liverpool, UK). The methodology and instrument were accredited by the international EQUIP standardisation programme [16]. Creatinine concentrations were evaluated using a standard Jaffe reaction-based microplate assay. A series of five quality control (QC) urines (along with a certified reference material (CRM) (Seronorm trace elements urine L-1)) were repeatedly analysed alongside participant samples to validate method accuracy. All QC and CRM categories remained within expected or certified ranges and displayed low variability.

For inter-laboratory comparison, a subset of BiB and validation samples (3.8%, 271 of 6971) were provided at regular intervals to the MRC Elsie Widdowson Laboratory in Cambridge. A high correlation was observed between UIC measurements across sites (*r* = 0.99). Full laboratory analysis details and validation protocols are provided in Additional file 1.

### Birth and pregnancy outcomes

The primary outcome was standardised birthweight (birthweight centile) calculated using UK customised growth charts (Gestation-Related Optimal Weight (GROW)) [17]. GROW calculations are valid and robust [18] and are used as part of standard growth assessment protocols in 81% of UK hospital trusts [19]. This approach calculates birthweight centile after considering maternal height, weight, parity, ethnicity, child’s sex, gestation length and birthweight (see Additional file 1 for further details). The following secondary birth and pregnancy outcomes were also examined: birthweight in grams, low birthweight (< 2.5 kg), SGA (< 10th centile), preterm birth (< 37 weeks), head circumference at birth, stillbirth and APGAR score measured at 1 and 5 min after birth.

### Intrauterine growth assessed via ultrasound

Foetal head circumference, biparietal diameter, femur length, abdominal circumference and estimated weight were measured via ultrasound scans amongst a sub-sample of BiB participants at 33 to 34 weeks’ gestation. Foetal weight was derived using the formula of Hadlock [20]. Scans were not offered to women with twins or known foetal abnormalities, and of 3805 women invited, 1859 scans were performed [21], with 1116 also providing urine samples.

### Assessment of congenital anomalies

Congenital anomalies were identified up to age 5 through careful examination of linked medical record data [22, 23]. Anomalies of metabolic origin (‘E70–90’ codes according to the 10th revision of the International Classification of Diseases) and genetic or chromosomal anomalies were excluded (details in Additional file 1).

### Statistical analysis

Urinary iodine-to-creatinine ratio (I:Cr) (μg/g) was used as the primary exposure to account for variation in urine dilution [24], though all analyses were also conducted using UIC. To avoid categorising iodine concentrations, restricted cubic splines were fitted with four knots placed at percentiles 5, 35, 65 and 95 and were used in all multiple linear and logistic regression models. Robust cluster (sandwich) estimates of variance were included in all models to account for heteroscedasticity and sibling clusters (successive pregnancies) [25].

Multiple imputation by chained equations, based on 100 imputed datasets, was used to correct for the effects of incomplete covariate information [26] (details in Additional file 1). Adjustment for confounding was informed by directed acyclic graphs (see Additional file 1: Figure S1). All models adjusted for maternal age, socioeconomic and education category (see details in Additional file 1: Table S1), ethnicity and season as potential confounders. Covariates were omitted, as appropriate, for modelling different outcomes (see Additional file 1: Table S2). Additional adjustments, for potential competing exposures, included smoking in pregnancy, alcohol in pregnancy, pregnancy complications (gestational diabetes, hypertension, preeclampsia), pre-pregnancy body mass index (BMI), parity, child’s sex and length of gestation. To adjust for seasonality, pairs of sine and cosine functions were prepared for each date across the year [27]. Models that only included confounders were not materially different from those additionally including competing exposures, and therefore, all reported results are from fully adjusted models.

Pre-specified sensitivity analyses (excluding iodine supplement users or those with pregnancy complications) and subgroup analyses (ethnic background or socioeconomic and education group) were undertaken for all outcomes (details in Additional file 1), except for binary outcomes where fewer than 100 cases were available. Additional sensitivity analyses were completed using dietary data available in a sub-sample of the cohort (see Additional file 1: Table S2). Figures present adjusted predicted outcomes (continuous variables) or probabilities (binary outcomes), across the range of iodine concentrations for an ‘average’ participant, i.e. primiparous, White European, non-smoker, who did not report consuming alcohol or experience complications in pregnancy, employed and not materially deprived, of mean gestation (39.6 weeks), age (27.2 years) and BMI (25.8 kg/m^2^). Predicted outcomes at the 25th and 75th percentiles of exposure were derived from these figures and tabulated.

The overall contribution of iodine status to each outcome was formally assessed with the Wald test. A two-tailed *p* value < 0.01 was considered statistically significant, and 99% confidence intervals (CIs) were used for all comparisons. Stata version 15.1 (StataCorp) was used for all analyses.

## Results

### Participant characteristics and iodine concentrations

In total, 12,453 women were recruited into BiB across 13,776 pregnancies; 6979 mothers (56%) provided a total of 7066 urine samples at 26–28 weeks’ gestation, relating to 7019 children, with some mothers participating during more than one pregnancy. Samples were then excluded where no linkage to child records could be made, for example, if the mother gave birth out of the area (*n* = 47), samples from twins and triplets (*n* = 339) and urine sample contamination or failed detection (*n* = 6). Analyses therefore included data from 6674 urine samples provided by 6355 mothers. Samples from pregnancies resulting in stillbirths (*n* = 37) were further excluded from all analyses except where ultrasound measurements or stillbirth was the outcome. A full overview of these exclusions is given in Additional file 1: Figure S2.

The median (interquartile range (IQR)) I:Cr was 83.1 μg/g (59.4 to 121.2), and UIC was 76.2 μg/L (44.6 to 120.2). UIC was < 50 μg/L in 2042 (29%) women, < 150 μg/L in 5925 (85%) women and exceeded 250 μg/L in just 236 (3%) women. For descriptive purposes, the cohort was split into 3 categories according to I:Cr. Women who were most economically deprived and less educated, those of Pakistani or other ethnic background, and those who used fewer supplements in pregnancy tended to have lower iodine levels (Table 1). In unadjusted observations, the children of mothers in the lowest I:Cr group tended to weigh less and were more likely to be SGA than those whose mothers had higher iodine concentrations (Table 2). In the lowest and highest I:Cr groups, the mean (standard deviation (SD)) birthweight centile was 42.0 (28.5) vs. 45.2 (28.6) and birthweight was 3217 g (542) vs. 3271 g (535), respectively. Pairwise correlations between the different growth measures are provided in Additional file 1: Table S3.

**Table 1:** Maternal characteristics according to urinary iodine-to-creatinine ratio

	All participants, *n* = 6637	Iodine-to-creatinine ratio (sample split into thirds)		
		Lower third (< 67 μg/g), *n* = 2213	Middle third (67 to 105 μg/g), *n* = 2212	Higher third (> 105 μg/g), *n* = 2212
I:Cr (μg/g), geometric mean (99% CI)	86.0 (84.5 to 87.5)	48.5 (47.8 to 49.3)	83.4 (82.8 to 84.0)	157.2 (154.3 to 160.2)
I:Cr (μg/g), median (IQR)	83.1 (59.4 to 121.2)	51.4 (42.7 to 59.4)	83.2 (74.4 to 93.3)	146.1 (121.2 to 185.2)
UIC (μg/L), geometric mean (99% CI)	70.8 (69.1 to 72.5)	45.9 (44.2 to 47.8)	70.0 (67.6 to 72.6)	110.4 (106.4 to 114.5)
UIC (μg/L), median (IQR)	76.2 (44.6 to 120.2)	52.2 (29.1 to 78.0)	77.4 (47.3 to 112.5)	117.6 (71.8 to 178.8)
Age (years), mean (SD)	27.2 (5.6)	26.5 (5.5)	27.3 (5.6)	27.8 (5.6)
BMI (kg/m^2^), mean (SD)	25.8 (5.5)	26.5 (5.8)	25.9 (5.4)	25.1 (5.1)
Socioeconomic status^†^				
Least deprived and most educated	1241 (21)	307 (16)	429 (22)	505 (25)
Employed, not materially deprived	1231 (21)	335 (17)	440 (22)	456 (23)
Employed, no access to money	917 (16)	320 (16)	295 (15)	302 (15)
Receives benefits, not materially deprived	1594 (27)	633 (32)	505 (26)	456 (23)
Most economically deprived	920 (16)	360 (18)	293 (15)	267 (13)
Ethnic background, *n* (%)				
White British and European	2877 (44)	800 (36)	1016 (46)	1061 (48)
Pakistani	2827 (43)	1064 (48)	914 (42)	849 (39)
Others (Black, Indian, mixed, others)	901 (14)	337 (15)	271 (12)	293 (13)
Parity				
0	2931 (44)	933 (42)	966 (44)	1032 (47)
1	1859 (28)	600 (27)	623 (28)	636 (29)
2	1059 (16)	371 (17)	361 (16)	327 (15)
3+	788 (12)	309 (14)	262 (12)	217 (10)
Health and lifestyle in pregnancy				
Gestational diabetes, *n* (%)	499 (8)	152 (7)	167 (8)	180 (8)
Pre-pregnancy hypertension, *n* (%)	50 (0.8)	21 (1.0)	17 (0.8)	12 (0.5)
Pregnancy-induced hypertension, *n* (%)	375 (6)	125 (6)	126 (6)	124 (6)
Preeclampsia, *n* (%)	173 (3)	61 (3)	55 (2)	57 (3)
Drank any alcohol, *n* (%)	958 (14)	299 (14)	335 (15)	324 (15)
Smoked, *n* (%)	969 (15)	302 (14)	352 (16)	315 (14)
Used any supplements, *n* (%)	1334 (20)	213 (10)	382 (17)	739 (33)
Iodine-containing supplements, *n* (%)	988 (15)	123 (6)	264 (12)	601 (27)
White fish intake* (g/day), mean (SD)	21.0 (27.0)	18.7 (26.3)	22.1 (28.2)	22.1 (26.1)
Oily fish intake* (g/day), mean (SD)	1.4 (3.8)	1.0 (3.2)	1.5 (4.2)	1.7 (4.0)
Total fish intake* (g/day), mean (SD)	23.8 (29.3)	20.8 (28.3)	25.1 (30.7)	25.4 (28.8)
Eat 5 fruits/vegetables per day*, *n* (%)				
Always	422 (19)	122 (16)	160 (21)	140 (19)
Sometimes	1700 (75)	601 (77)	558 (72)	541 (75)
Never	151 (7)	57 (7)	52 (7)	42 (6)

*BMI* body mass index, *CI* confidence intervals, *I:Cr* urinary iodine-to-creatinine ratio, *IQR* interquartile range, *SD* standard deviation, *UIC* urinary iodine concentration

*Data from women who were asked about diet in pregnancy (*n* = 2776)

^†^Refer to supplement table S1 for details

**Table 2:** Pregnancy and birth outcomes for all participants and according to maternal iodine-to-creatinine ratio

	Iodine-to-creatinine ratio (cohort split into thirds)			
	All participants, *n* = 6637	Lower third (< 67 μg/g), *n* = 2213	Middle third (67 to 105 μg/g), *n* = 2212	Higher third (> 105 μg/g), *n* = 2212
Size at birth				
Birthweight centile, mean (SD)	43.6 (28.5)	42.0 (28.1)	43.7 (28.9)	45.2 (28.6)
Birthweight (g), mean (SD)	3245 (549)	3217 (542)	3247 (568)	3271 (535)
Small for gestational age (< 10th centile), *n* (%)	880 (13)	311 (14)	298 (13)	271 (12)
Low birthweight (< 2.5 kg), *n* (%)	466 (7)	167 (8)	161 (7)	138 (6)
Head circumference at birth (mm), mean (SD)	343 (16)	343 (15)	343 (17)	344 (15)
APGAR score (out of 10), median (IQR)				
1 min	8.5 (1.4)	8.5 (1.3)	8.4 (1.4)	8.5 (1.1)
5 min	9.0 (0.7)	9.0 (0.7)	9.0 (0.7)	9.1 (0.7)
Ultrasound scan (34 weeks) estimations, mean (SD)				
Head circumference (mm)	315 (9)	314 (9)	315 (9)	315 (9)
Biparietal diameter (mm)	8.5 (0.3)	8.5 (0.3)	8.5 (0.3)	8.5 (0.3)
Femur length (mm)	65 (2)	65 (2)	65 (2)	65 (2)
Abdominal circumference (mm)	295 (14)	294 (14)	295 (14)	296 (14)
Estimated weight (g)	2237 (237)	2228 (244)	2234 (228)	2248 (240)
Preterm (< 37 weeks), *n* (%)	354 (5)	114 (5)	134 (6)	106 (5)
Stillbirth, *n* (%)	37 (0.6)	12 (0.5)	14 (0.6)	11 (0.5)
Congenital anomalies, *n* (%)				
All	353 (5.3)	123 (5.6)	127 (5.7)	103 (4.7)
Nervous system	125 (1.9)	39 (1.8)	44 (2.0)	42 (1.9)

### Birth and pregnancy outcomes

**Fig. 1:**
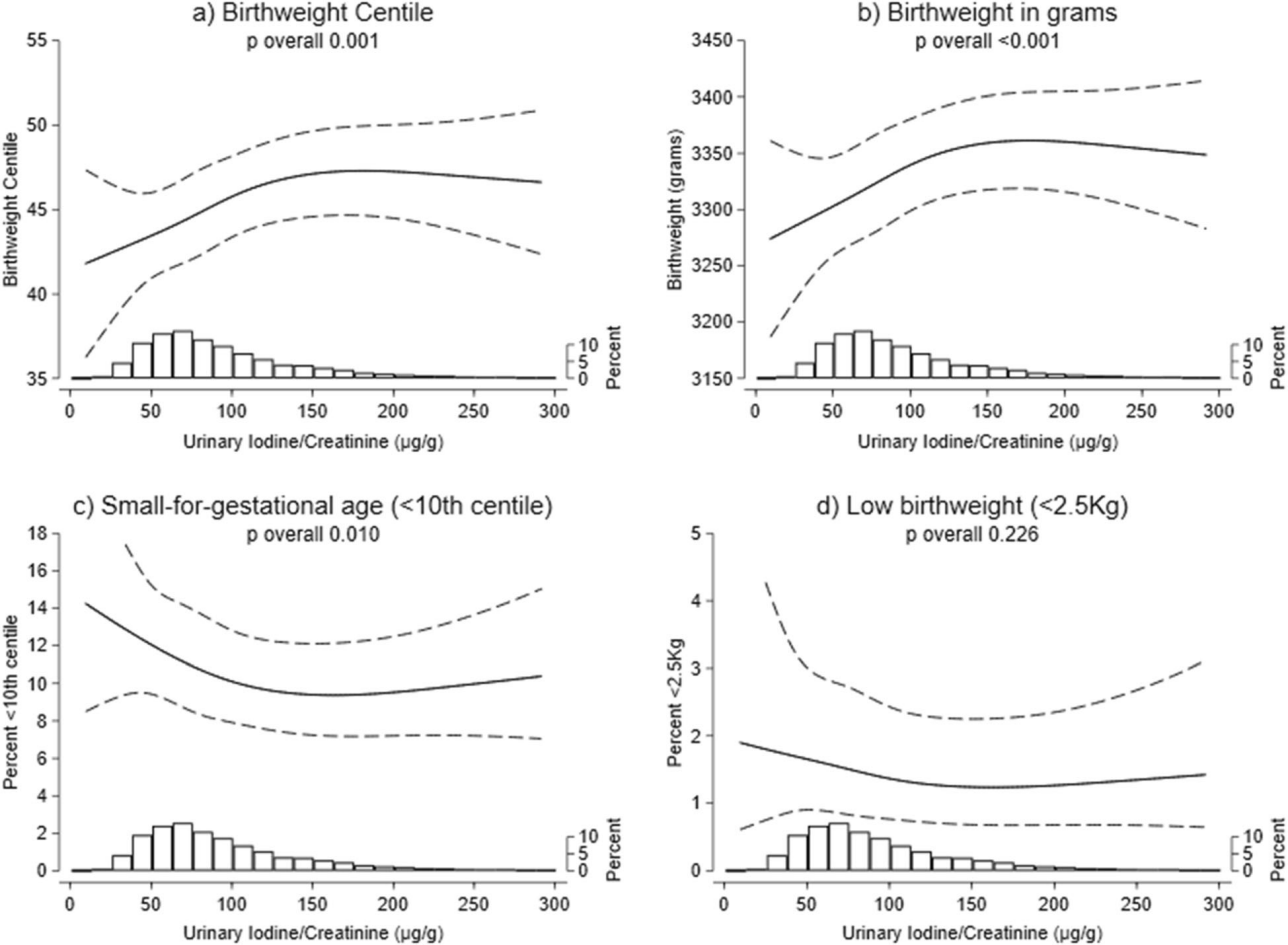
Estimated birth size for children of typical mothers, across the range of maternal I:Cr concentrations. Histograms illustrate the distribution of iodine concentrations, and although the figures are curtailed at 300 μg/g, the splines (solid line) and 99% CIs (dashed lines) were drawn using data from all participants. Splines were drawn after adjustment for confounders (details in Supplemental table S1). The spline position in these figures illustrates the predicted estimate for typical participants (primiparous; White ethnic background; ‘employed and not materially deprived’; did not smoke, drink or experience complications in pregnancy; and have average BMI, age and gestation length)

There was evidence of a positive association between I:Cr and both birthweight centile and birthweight in grams in adjusted analyses (Fig. 1a–c, Additional file 1: Table S4). For a mother with typical characteristics (defined above), the birthweight centile at the 25th percentile of I:Cr (59 μg/g) was 2.7 percentage points lower than that at the 75th percentile of I:Cr (121 μg/g) (99% CI 0.8 to 4.6, *p*_overall_ 0.001). Similarly, predicted birthweight was 41 g lower at the 25th I:Cr percentile than at the 75th (99% CI 13 to 69, *p*_overall_ 0.001). The probability of SGA was also 1.9 percentage points higher (99% CI 0.0 to 3.7, *p*_overall_ 0.010). However, there was no evidence of an association for low birthweight (< 2.5 kg) (Fig. 1d, Additional file 1: Table S4). Apparent thresholds in the association between I:Cr and birthweight centile, birthweight in grams and SGA around 150 μg/g (Fig. 1) should be interpreted with caution given the small proportion of participants with I:Cr measurements above this level (15%), with associated wide CIs and inconsistency of thresholds in sensitivity analyses (below).

**Fig. 2:**
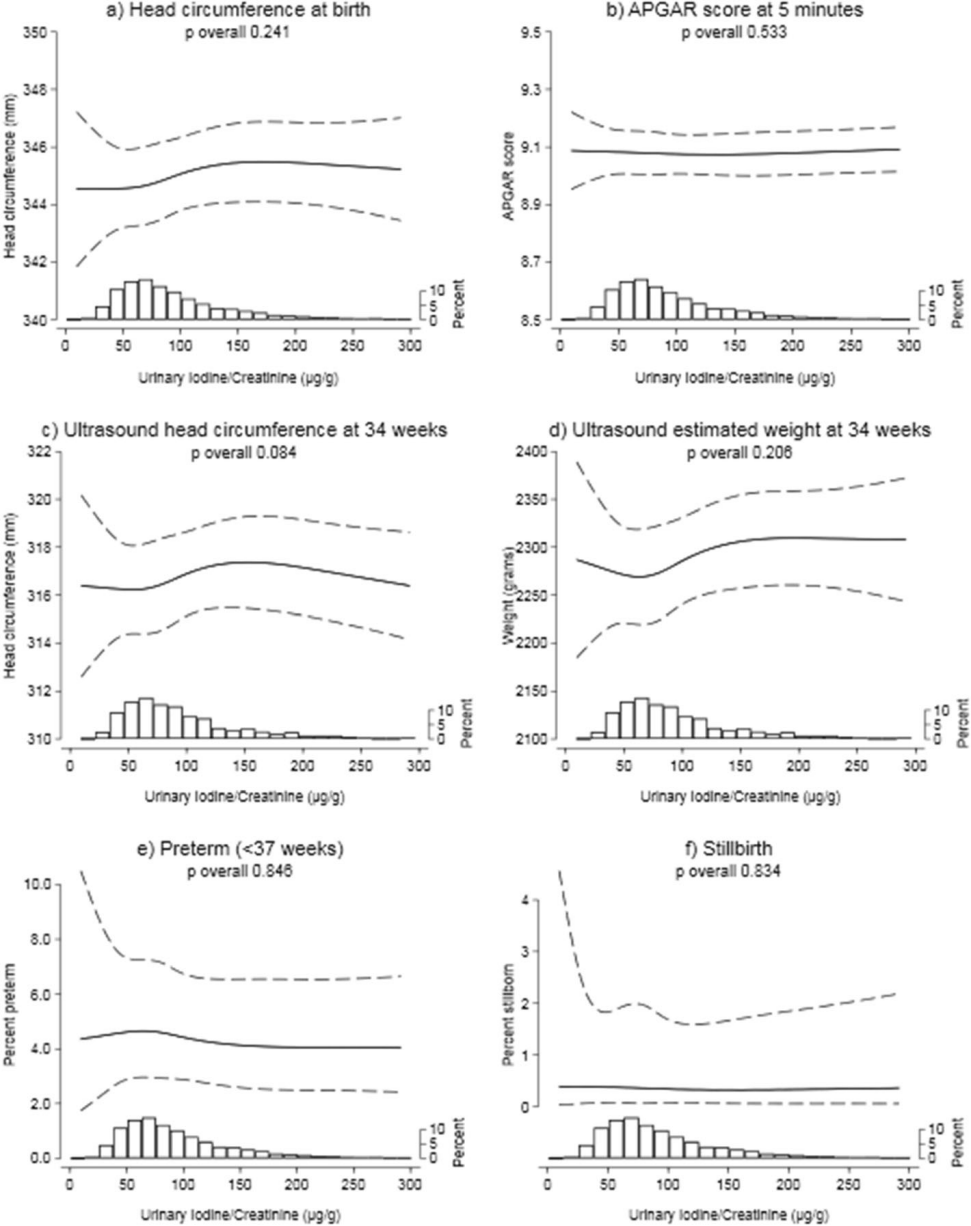
Estimated intrauterine size and pregnancy outcomes for typical mothers, across the range of maternal I:Cr concentrations. Histograms illustrate the distribution of iodine concentrations, and although the figures are curtailed at 300 μg/g, the splines (solid line) and 99% CIs (dashed lines) were drawn using data from all participants. Splines were drawn after adjustment for confounders (details in Supplemental table S1). The spline position in these figures illustrates the predicted estimate for typical participants (primiparous; White ethnic background; ‘employed and not materially deprived’; did not smoke, drink or experience complications in pregnancy; and have average BMI, age and gestation length)

There was no evidence of any association between I:Cr and ultrasound measurements at 34 weeks’ gestation, head circumference at birth or APGAR score measured at 1 or 5 min after birth. Similarly, the probability of stillbirth, preterm birth or congenital anomalies was also not associated with maternal iodine concentration (Fig. 2, Additional file 1: Figure S3 & Table S4). There was no evidence of an association between UIC and the outcomes examined (Additional file 1: Table S4).

### Subgroup and sensitivity analyses

There was no evidence of effect modification (interaction) by ethnic background (White European vs. Pakistani) in the association between I:Cr and any outcome (size at birth, intrauterine size, APGAR score, preterm or congenital anomalies). For the comparison between socioeconomic and education categories (more deprived and less educated vs. less deprived and more educated), there was only evidence of effect modification in the association between I:Cr and birthweight centile (*p* = 0.008) and head circumference at birth (*p* = 0.007) (Additional file 1: Figure S5 & S10, panel g-h). In the more deprived and less educated women, there was a stronger tendency for higher birthweight centile with higher I:Cr across the range of usual intakes (note frequency histograms). Differences in association patterns were less obvious for head circumference.

Estimates from a sensitivity analysis using only women with complete data were not substantively different from primary analyses using imputed data (Additional file 1: Figure S5-S14, panel a). For all outcomes, sensitivity analyses (excluding outliers, those with complications in pregnancy or iodine supplement users) were not materially different from the full cohort analyses (Additional file 1: Figure S5-S14, panel b-d). Apparent thresholds in the association between I:Cr and birthweight centile, SGA and birthweight in grams, around 150 μg/g (Fig. 1) were no longer present after excluding a small number of I:Cr outliers (*n* = 39) (Additional file 1: Figure S5-S7, panel b), with trends remaining similar across the range of I:Cr values where more data were available. There was no evidence of any association after additionally adjusting for diet in pregnancy (data available for approximately one third of participants). Results for congenital anomalies were not different after excluding consanguineous relationships.

## Discussion

In the largest study of its type to date, and in a pregnant population characterised as having insufficient iodine intake according to WHO-outlined thresholds (UIC < 150 μg/L), evidence was found to support a small association between lower I:Cr measured at 26–28 weeks’ gestation and lower birthweight centile, birthweight in grams and higher probability of SGA. In absolute terms, between the 25th and 75th I:Cr percentile (59 and 121 μg/g), birthweight centile was estimated to be 2.7% higher, birthweight was 41 g higher and the probability of being SGA was 1.9% lower, for a typical participant in this cohort. The estimated birthweight difference of 41 g is small but of comparable size to the birthweight differences observed with environmental tobacco smoke exposure in pregnancy [28]. There was no evidence to support associations between I:Cr and all other outcomes examined, including measures of intrauterine growth from ultrasound scans at 34 weeks’ gestation, head circumference at birth, APGAR score, low birthweight, stillbirth, preterm birth and congenital anomalies. Additionally, UIC was not found to be associated with any outcomes.

Amongst comparable studies, this is the largest to date, with the next largest study including 3140 pregnancies [11]. Our findings for birthweight are concordant with three prior studies reporting associations of lower birthweight with lower iodine status [4, 5, 7]. However, in each case, the association was only examined with UIC, but UIC was not associated with any outcomes in this study. Six other studies also report no evidence of associations between birthweight and UIC [6, 8–12] or I:Cr [11, 12]. The higher probability of SGA with lower I:Cr observed here also supports observations from one prior study [4], but not five others reporting no evidence of associations [6, 7, 10–12]. Inconsistency between studies and within specific outcomes may be attributed to differences in the time period of assessment, with iodine status during key developmental stages potentially having differential effects on foetal growth. Furthermore, only three prior studies corrected for urinary dilution using creatinine concentration in urine. The absence of associations across all birth or pregnancy outcomes in this study may reflect measurement error in some outcome assessments such as for intrauterine growth. Alternatively, iodine status at 26–28 weeks’ gestation may be outside of critically important time windows for some aspects of development, or iodine status may not be responsible for the effects on these birth or pregnancy outcomes.

In this study, a sampling point of 26–28 weeks was selected to ensure iodine exposure assessment was conducted before the ultrasound outcomes measured at around 34 weeks. Foetal iodine demands are known to increase throughout pregnancy [29], with demands peaking during the second half of pregnancy [30]. However, despite this increase in demand, iodine excretion has been demonstrated to remain somewhat constant throughout the pregnancy in other populations presenting mild iodine deficiency [31, 32]. Our time point therefore likely represents a snapshot of a pregnant population during a period of peak thyroid stress and of stable iodine excretion.

According to the WHO-outlined threshold [3], pregnant populations with sufficient iodine have a median UIC > 150 μg/L, but the median UIC in this study was just 76 μg/L. Whilst observing a wide range of iodine concentrations in the BiB cohort, median UIC was the lowest of all previous studies [4–12]. The difference potentially positions this work to better quantify associations in lower sufficiency settings. Higher concentrations in some settings may explain why several previous studies did not report associations for key growth outcomes, as any effect of iodine is likely to be dose-dependent. The effect sizes reported here are dependent on the iodine distribution in the BiB study population, and replication is therefore warranted in other settings.

In the UK, where salt iodisation or supplementation for iodine is not routine, iodine is primarily sourced from only a few foods (largely from milk but also from some fish, meat and cereals) [33]. Women who avoid dairy or are vegetarian and who do not use supplements may therefore be at greater risk of insufficiency, particularly during pregnancy when iodine demands rise, and consequently may be at greater risk of having lower-weight babies. Lower weight at birth is a well-established risk factor for chronic disease in later life [13, 34]. Furthermore, some women may be at greater risk of having low iodine status: the UK Low Income National Diet and Nutrition Survey reported that Black or Asian women were more likely to be below the UK’s lower reference nutrient intake than White women [35]. The predicted estimates in this study may also be amplified amongst those with several risk factors for smaller-birthweight babies. The majority of women of reproductive age are likely unaware of their iodine status or good dietary sources, as only 23% of women who were pregnant or of reproductive age in the UK had heard of iodine, compared to 100% for folic acid [36]. Given the presence of these at-risk groups, and evidence of a continuing decrease in iodine consumption amongst UK women of childbearing age [35], our finding of an association between lower iodine status and increased risk of lower birthweight is highly relevant for the UK population.

There was evidence of different associations between I:Cr and birthweight centile in women according to deprivation and education category, with more apparent associations in those who were ‘more deprived and less educated’. These differences may result from residual confounding or could indicate better resilience or some compensatory mechanisms amongst more affluent and educated women, possibly driven by generally better general health or dietary status, and thus, women who are ‘less educated and more deprived’ may be at greater risk of potential negative effects of lower iodine status in pregnancy.

Severe iodine deficiency results in a range of disorders in offspring, including mental deficiency and short stature [3]. Iodine plays an essential role in thyroid-mediated foetal growth and development [2], but there is little evidence to indicate an optimum iodine concentration or a threshold associated with better pregnancy or birth outcomes. Within the range of comparatively low iodine concentrations observed in this study, evidence for a threshold or plateau in associations was weak.

Study strengths include the large study size and power to detect potential differences in associations. Data are from a well-characterised and multi-ethnic cohort with a comprehensive range of objective outcomes, allowing for evaluation of potential associations with different aspects of growth and development in utero and at birth. A more accurate measure of iodine status in pregnant women was achieved by accounting for urine dilution variability using I:Cr [37]. Spot urine samples are considered a reliable marker of population iodine status by the WHO [3], and all samples used here were collected after overnight fasting and at a similar time of day. The robust and validated urine sample analysis, including participation in the international EQUIP programme [16], is a strength in this work. The use of standardised birthweights, calculated using nationally representative datasets, is also a strength, along with data-driven exploration of potential associations and threshold effects, rather than the arbitrary categorisation of iodine concentration. Participants were drawn from one geographic region but covered a wide range of social backgrounds and different ethnicities. However, in sensitivity analyses, our results are consistent across various subgroups suggesting generalizability.

Despite carefully controlling for potential confounders, there remains the chance that observed associations result from residual confounding in unmeasured or inaccurately characterised variables. The single assessment of urinary iodine also limits the exploration of changes through pregnancy and any possible effects on stages of foetal development. Random measurement error in estimating iodine status from single spot urine samples may also limit analyses; however, this would bias estimates towards the null [38] and may only explain why associations were not seen for other outcomes.

It remains challenging to establish whether the absence of associations in some other studies resulted from measurement error, assessment during less critically important time periods in pregnancy or the fact that other populations had higher median UIC and may therefore have been less likely to observe associations. Further studies in populations without routine fortification programmes or with relatively low status or that evaluate birth outcomes in relation to iodine status throughout pregnancy (or prior to pregnancy) may provide clarity on these issues. Research into alternative biomarkers will also help to improve characterisation of usual or changes in maternal iodine status.

## Conclusions

Many countries lack pregnancy-specific recommendations relating to iodine intake or iodine status, owing to the absence of strong or consistent evidence, at least amongst those without severe deficiency. Given our finding of an association between lower iodine status and lower birthweight, along with the low status of women of reproductive age in this and other countries in Europe [12, 39], this knowledge gap should be addressed as a priority, particularly as lower birthweight has been linked to increased incidence of diabetes [40], risk of leukaemia [41] and all-cause adult mortality [13, 34].

## Supplementary information

**Additional file 1: Table S1.** Details of socioeconomic position categories and maternal education levels. **Table S2.** Details of model covariates, sample exclusions, sensitivity analyses and subgroup analyses for each of the different outcomes. **Table S3.** Pairwise correlations between each of the growth measures. **Table S4.** Predicted estimates (continuous outcomes) and percent at the threshold (binary outcomes) (99% CIs) at the 25th, 50th and 75th centiles of iodine concentration and p-_overall_* for ‘average’ participants†. **Figure S1.** A directed acyclic graph used to identify confounders and competing exposures in the association between maternal iodine concentration and birth or pregnancy outcomes. **Figure S2.** Flow chart of participant inclusions and exclusions. **Figure S3.** Estimated APGAR score at 1 minute and percent with congenital anomalies, using imputed datasets for the full sample. **Figure S4.** Estimated biparietal diameter, femur length and abdominal circumference, from ultrasound scans at 34 weeks’ gestation, across the range of maternal I:Cr concentrations using imputed datasets for the full sample. **Figure S5.** Estimated birthweight centile in all participants, sensitivity analysis and subgroups. **Figure S6.** Estimated birthweight in grams in all participants, sensitivity analysis and subgroups. **Figure S7.** Probability of being small for gestational age in all participants, sensitivity analysis and subgroups. **Figure S8.** Probability of low birthweight in all participants, sensitivity analysis and subgroups. **Figure S9.** Apgar score at 5 minutes after birth in all participants, sensitivity analysis and subgroups. **Figure S10.** Estimated head circumference at birth in all participants, sensitivity analysis and subgroups. **Figure S11.** Estimated head circumference from 34 week ultrasound scan in all participants, sensitivity analysis and subgroups. **Figure S12.** Estimated weight from 34 week ultrasound scan in all participants, sensitivity analysis and subgroups. **Figure S13.** Probability of preterm birth in all participants, sensitivity analysis and subgroups. **Figure S14.** Probability of congenital anomalies in all participants, sensitivity analysis and subgroups.

## Data Availability

Born in Bradford welcomes collaboration with other researchers. Requests for existing data and biological samples will be reviewed, prioritised and authorised by the BiB Executive Group. Potential collaborators should complete an outline proforma available on the Born in Bradford website (borninbradford.nhs.uk) and submit to the BiB Director.

## Abbreviations

BiB: Born in Bradford
BMI: Body mass index
CIs: Confidence intervals
CRM: Certified reference material
GROW: Gestation-Related Optimal Weight
I:Cr: Urinary iodine-to-creatinine ratio
IQR: Interquartile range
QC: Quality control
SD: Standard deviation
SGA: Small for gestational age
UIC: Urinary iodine concentration
WHO: World Health Organization
