# Supplementary material for: Maternal iodine status, intrauterine growth, birth outcomes and congenital anomalies in a UK birth cohort
Source: BMC Med. 2020 Jun 11;18:132. doi: 10.1186/s12916-020-01602-0 (PMC7288513; doi:10.1186/s12916-020-01602-0)
Supplement: Supplementary file 1 — Additional file 1: Table S1. Details of socioeconomic position categories and maternal education levels. Table S2. Details of model covariates, sample exclusions, sensitivity analyses and subgroup analyses for each of the different outcomes. Table S3. Pairwise correlations between each of the growth measures. Table S4. Predicted estimates (continuous outcomes) and percent at the threshold (binary outcomes) (99% CIs) at the 25th, 50th and 75th centiles of iodine concentration and p-overall* for ‘average’ participants†. Figure S1. A directed acyclic graph used to identify confounders and competing exposures in the association between maternal iodine concentration and birth or pregnancy outcomes. Figure S2. Flow chart of participant inclusions and exclusions. Figure S3. Estimated APGAR score at 1 minute and percent with congenital anomalies, using imputed datasets for the full sample. Figure S4. Estimated biparietal diameter, femur length and abdominal circumference, from ultrasound scans at 34 weeks’ gestation, across the range of maternal I:Cr concentrations using imputed datasets for the full sample. Figure S5. Estimated birthweight centile in all participants, sensitivity analysis and subgroups. Figure S6. Estimated birthweight in grams in all participants, sensitivity analysis and subgroups. Figure S7. Probability of being small for gestational age in all participants, sensitivity analysis and subgroups. Figure S8. Probability of low birthweight in all participants, sensitivity analysis and subgroups. Figure S9. Apgar score at 5 minutes after birth in all participants, sensitivity analysis and subgroups. Figure S10. Estimated head circumference at birth in all participants, sensitivity analysis and subgroups. Figure S11. Estimated head circumference from 34 week ultrasound scan in all participants, sensitivity analysis and subgroups. Figure S12. Estimated weight from 34 week ultrasound scan in all participants, sensitivity analysis and subgroups. Figure S13. Proba [file 12916_2020_1602_MOESM1_ESM.docx]

**Additional file 1**

**Maternal iodine status, intrauterine growth, birth outcomes and congenital anomalies in a UK birth cohort**

**Additional laboratory analysis details**

Sample concentration was determined against a urine matrix matched standard curve spiked with 0, 5, 10, 40, 70, 100, 400 and 800µg/L iodide (Sigma Aldrich).

A series of quality control (QC) urines with previously determined UIC ranges were analysed alongside participant samples. The first series comprised of QC urine A (target iodine value: 71·6µg/L, 60.2 – 80.1), B (target iodine value: 103·6µg/L, 87·5 – 118·9) and C (target iodine value: 164·1µg/L, 133·4 – 189·8). The second series replaced QC urine A with QC urine D (target iodine value: 56·8µg/L, 44·8 – 66·4) due to QC urine depletion. The third series replaced QC urine C with QC urine E (target iodine value: 155·6µg/L, 139·7 – 173·4), again due to depletion. Target urine values and ranges were determined via the three step procedure outlined in the CDC EQUIP standardisation programme. Participant samples, quality control samples, internal standards and certified reference materials were vortexed, centrifuged, and diluted 1:10 before analysis. Final samples consisted of 500 µL of urine, 4000µL of diluent (1% tetramethylammonium hydroxide (Sigma Aldrich), 0.01% Triton X-100 (Sigma Aldrich)). Diluent was spiked with 10 µg/L of tellurium to act as an internal ICP-MS reference standard. A certified reference material (Seronorm Trace Metal Urine Level 1) was included in each run to provide external validation (target iodine value 105µg/L, certified range: 84-126).

Observed mean values for QC urines across the course of the study were 70·7μg/L (n=99), 103·2μg/L (n=410) and 161·6μg/L (n=150) for urines A, B and C respectively. The observed values for QC urines D and E were 55.6μg/L (n=329) and 156.5μg/L (n=261). Certified reference material gave a mean value of 107.7μg/L across all runs. Assessment of intra-run precision for QC samples gave coefficients of variation (CV) of 1.03% at 71.5μg/L (A), 2.43% at 101.3μg/L (B), 1.57 at 160.1μg/L (C), 2.67 at 58.0μg/L (D) and 1.06 at 145.0μg/L (E). Inter-run precision measures for QC samples gave a CV of 7.5% at 70.7μg/L (A), 7.6% at 103.2μg/L (B), 8.7% at 161.6μg/L (C), 9.7% at 55.6μg/L (D) and 5.4% at 156.5μg/L (E). Assessment of intra-run precision for certified reference material gave a CV of 1.76% at 106.4μg/L. Assessment of inter-run precision for certified reference material gave a CV of 5.5% at 107.7μg/L.

The method limit of quantification was 1.46μg/L.

Urinary creatinine concentrations were assessed through a standard microplate assay utilising the Jaffe reaction. Assessment of creatinine intra-run precision gave a CV of 2% at 10 mg/L, 1% at 70mg/L and 1.1% at 120mg/L. Assessment of inter-run precision gave a CV of 14.5% at 10mg/L, 9.7% at 70mg/L and 5.5% at 120mg/L.

**Use of GROW calculator for birthweight centile**

Birthweight centiles were developed by the National Perinatal Epidemiology Unit in Oxford, based on over 1 million births in England and Wales as part of the national perinatal surveillance programme, the model was then validated against over 600 thousand births.^33^

For any missing required variables, the GROW calculator applies mean UK values (e.g. missing maternal weight) but since this may be inappropriate for women of Pakistani or other ethnic background, the mean height and weight of ethnic groups within BiB were calculated and these values were applied to the women with missing height (n=2535/13957) or weight data (n=201/13957) before centile calculation.

**Congenital anomaly data**

Cases and diagnoses made in the first year of life were reviewed by systematic case note review.^21^ Subsequent cases were identified from diagnosis codes reported in primary care records and were reviewed by a clinical geneticist.^22^ Anomalies were identified and grouped into system or anomaly sub-types according to standard guidelines from European (EUROCAT) and British (BINOCAR) congenital anomaly registers.^22^ Congenital anomaly sub-types were separately examined where a minimum of 100 cases were available.

**Subgroup and sensitivity analysis**

Sensitivity analyses were also conducted to assess robustness of results: (i) excluding extreme iodine concentrations (i.e. outside 3 standard deviations (SD) of the mean on the log scale) (n=39), (ii) excluding women with complications in pregnancy (gestational diabetes, hypertension or preeclampsia) (n=899), and (iii) to exclude users of iodine-containing supplements (n=988). Consanguineous relationships (any known blood relationship between parent participants or their respective parents) were also excluded in a sensitivity analysis for the congenital anomaly outcomes (n=1683). Models were also run separately by ethnic group (White or Pakistani ethnic background) and by maternal socioeconomic and education category ('more deprived and less educated' or 'less deprived and more educated' (supplement Table S2)). Though socioeconomic and education category was included in its five original categories for main analysis, for the purpose of formally testing subgroup interaction, two categories were used.

**Multiple imputation details**

Multiple imputation by chained equations, based on 100 imputed datasets, was used to correct for the effects of incomplete covariate information.^25^ All model covariates were used in imputation, including the outcome and iodine splines. Separate imputation models were used for each outcome and for analyses using UIC or I:Cr. Note that variables used in calculating birthweight centiles (maternal height, weight, parity, child's sex, gestation length and ethnicity) were not included in imputation models for this outcome. Predictive mean matching was used for continuous variables and logistic regression for categorical.

Missing data imputed:

| **Variable** | **Missing data out of 6637 pregnancies*** |
| --- | --- |
| BMI | 826 |
| Ethnicity | 32 |
| Socioeconomic & education category | 734 |
| Preeclampsia | 317 |
| Pregnancy-induced hypertension | 304 |
| Pre pregnancy hypertension | 308 |
| Gestational diabetes | 6 |
| Smoking status | 722 |
| Alcohol use in pregnancy | 757 |

*Note that missing data numbers varied for each imputation model according to the presence of relevant outcomes. These numbers relate to the whole sample.

**Table S1:** Details of socioeconomic position categories and maternal education levels

| N (%) | | **Less deprived and more educated**  (derived using three categories from latent class analysis*   1. Least socioeconomically deprived and most educated (n=1241), 2. Employed and not materially deprived (n=1231), 3. Employed and no access to money (n=917)) | **More deprived and less educated**  (derived using two categories from latent class analyses*   1. Receiving benefits and not materially deprived (n=1594), 2. Most economically deprived (n=920)) |
| --- | --- | --- | --- |
| Highest maternal educational achievement | |  |  |
|  | <5 GCSE equivalent | 139 (4) | 1075 (43) |
|  | 5 GCSE equivalent | 801 (24) | 937 (37) |
|  | A-level equivalent | 700 (21) | 183 (7) |
|  | Higher than A-level | 1440 (42) | 169 (7) |
|  | Don't know/ other | 309 (9) | 150 (6) |
|  |  |  |  |
| In receipt of means-tested benefits | | 632 (19) | 1672 (67) |

*The 5 original socioeconomic position categories were derived from latent class analysis, to group women with similar socioeconomic position profiles.^34^ To do this, 19 determinants were included in the analysis, including maternal and paternal employment, maternal and paternal education, receipt of benefits, housing tenure and the ability to save or buy goods and services. These 5 categories were then divided into two groups to approximately represent women who were 'Less deprived and more educated' or 'More deprived and less educated'.

**Table S2:** Details of model covariates, sample exclusions, sensitivity analyses and subgroup analyses for each of the different outcomes

|  |  |  |  | **OUTCOMES** | | | | | |
| --- | --- | --- | --- | --- | --- | --- | --- | --- | --- |
| **Models** | | |  | **• Standardised birthweight (centiles) ^1^,**  **• SGA (under 10^th^ Centile)^1^** | **• Birthweight (grams),**  **• LBW (<2500g),**  **• Head circumference,**  **• APGAR score (1m & 5m)** | **• Preterm (<37 weeks),**  **• Stillbirth** | **Ultrasound measures:**  **• Head circumference,**  **• Estimated fetal weight** | **Secondary ultrasound measures:**  **• Biparietal diameter,**  **• Femur length,**  **• Abdominal circumference** | **• Congenital anomalies** |
| **M1** | **M2** | **M3** | **Model covariates** |  |  |  |  |  |  |
| ✓ | ✓ | ✓ | Maternal age, years | ✓ | ✓ | ✓ | ✓ | ✓ | ✓ |
| ✓ | ✓ | ✓ | Socioeconomic and education category^3^ | ✓ | ✓ | ✓ | ✓ | ✓ | ✓ |
| ✓ | ✓ | ✓ | Ethnicity (White, Pakistani, Other) |  | ✓ | ✓ | ✓ | ✓ | ✓ |
| ✓ | ✓ | ✓ | Season^6^ | ✓ | ✓ | ✓ | ✓ | ✓ | ✓ |
|  | ✓ | ✓ | BMI (pre-pregnancy), Kg/M^2^ |  | ✓ | ✓ | ✓ | ✓ | ✓ |
|  | ✓ | ✓ | Parity (0, 1, 2, 3+) |  | ✓ | ✓ | ✓ | ✓ | ✓ |
|  | ✓ | ✓ | Smoked in pregnancy (yes/no) | ✓ | ✓ | ✓ | ✓ | ✓ | ✓ |
|  | ✓ | ✓ | Alcohol in pregnancy (yes/no) | ✓ | ✓ | ✓ | ✓ | ✓ | ✓ |
|  | ✓ | ✓ | Gestational diabetes (yes/no) | ✓ | ✓ | ✓ | ✓ | ✓ | ✓ |
|  | ✓ | ✓ | Hypertension (pre-pregnancy or pregnancy-induced) (yes/no) | ✓ | ✓ | ✓ | ✓ | ✓ | ✓ |
|  | ✓ | ✓ | Pre-eclampsia (yes/no) | ✓ | ✓ | ✓ | ✓ | ✓ | ✓ |
|  | ✓ | ✓ | Child's sex |  | ✓ | ✓ | ✓ | ✓ | ✓ |
|  | ✓ | ✓ | Length of gestation (days) |  | ✓ |  | ✓ | ✓ | ✓ |
|  |  | ✓ | Total fish intake (g/week) | ✓ | ✓ | ✓ | ✓ | ✓ | ✓ |
|  |  | ✓ | Eats 5 fruit/vegetables per day (Never, sometimes, always) | ✓ | ✓ | ✓ | ✓ | ✓ | ✓ |
|  |  |  | **Sample exclusions** |  |  |  |  |  |  |
|  |  |  | Stillbirths | ✓ | ✓ |  | ✓ |  | ✓ |
|  |  |  | Twins/Triplets | ✓ | ✓ | ✓ | ✓ |  | ✓ |
|  |  |  | **Sensitivity analysis** |  |  |  |  |  |  |
|  |  |  | Exclude extreme iodine values | ✓ | ✓ | ✓ | ✓ |  | ✓ |
|  |  |  | Exclude iodine-containing supplement users | ✓ | ✓ | ✓ | ✓ |  | ✓ |
|  |  |  | Exclude pregnancy complications (diabetes, hypertension, pre-eclampsia) | ✓ | ✓ | ✓ | ✓ |  | ✓ |
|  |  |  | Exclude consanguineous relationships |  |  |  | ✓ |  | ✓ |
|  |  |  |  |  |  |  |  |  |  |
|  |  |  | **Subgroup analysis** |  |  |  |  |  |  |
|  |  |  | Ethnic group (White & Pakistani origin) | ✓ | ✓ | ✓ preterm only^5^ | ✓ |  | ✓ |
|  |  |  | Socioeconomic and education position in 2 categories^4^ | ✓ | ✓ | ✓ preterm only^5^ | ✓ |  | ✓ |

^1.^ Centiles calculated using data on maternal height and weight, parity, ethnicity, child's sex and gestation length (these covariates therefore omitted)

^2.^ Length of gestation at time of ultrasound scan

^3.^ Five categories of socioeconomic and education position^34^ (see Table S2 for further details)

^4.^ Two categories of socioeconomic and education position (see Table S2 for further details)

^5.^ Subgroup analyses not conducted where case numbers were too small.

^6.^ Season of urine sample collection was included in all models because of the known associations with iodine status,^35^ birthweight or development in UK populations^36^ and congenital anomalies in Europe.^37^

M1: Confounders identified using DAG

M2: Full model including confounders and competing exposures. These results are reported in manuscript.

M3: Sensitivity analysis in cohort subgroup with dietary data available (n=2776) in a subsample of the cohort. Additional adjustments included total fish intake (g/day) and consuming five portions of fruit and vegetables per day (never/ sometimes/ always)

**Table S3**: Pairwise correlations between each of the growth measures

|  | Birthweight centile | Birthweight grams | Birth head circumference | Apgar score at 1 minute | Apgar score at 5 minutes | Ultrasound head circumference | Ultrasound biparietal diameter | Ultrasound femur length | Ultrasound abdominal circumference | Ultrasound estimated weight |
| --- | --- | --- | --- | --- | --- | --- | --- | --- | --- | --- |
| Birthweight centile | 1.00 |  |  |  |  |  |  |  |  |  |
| Birthweight grams | 0.71 | 1.00 |  |  |  |  |  |  |  |  |
| Birth head circumference | 0.44 | 0.74 | 1.00 |  |  |  |  |  |  |  |
| Apgar score at 1 minute | -0.04 | 0.02 | -0.01 | 1.00 |  |  |  |  |  |  |
| Apgar score at 5 minutes | 0.01 | 0.08 | 0.04 | 0.51 | 1.00 |  |  |  |  |  |
| Ultrasound head circumference | 0.34 | 0.43 | 0.54 | -0.07 | 0.01 | 1.00 |  |  |  |  |
| Ultrasound biparietal diameter | 0.33 | 0.41 | 0.50 | -0.06 | 0.00 | 0.72 | 1.00 |  |  |  |
| Ultrasound femur length | 0.35 | 0.35 | 0.26 | -0.06 | 0.01 | 0.37 | 0.27 | 1.00 |  |  |
| Ultrasound abdominal circumference | 0.50 | 0.54 | 0.43 | -0.07 | 0.02 | 0.58 | 0.44 | 0.42 | 1.00 |  |
| Ultrasound estimated weight | 0.52 | 0.56 | 0.48 | -0.08 | 0.02 | 0.72 | 0.53 | 0.65 | 0.94 | 1.00 |

**Table S4**: Predicted estimates (continuous outcomes) and percent at the threshold (binary outcomes) (99% CIs) at the 25^th^, 50^th^ and 75^th^ centiles of iodine concentration and p-_overall_* for 'average' participants†

|  |  | **Iodine to creatinine ratio (µg/g)** | | | | | **Urinary iodine concentration (µg/L)** | | | | |
| --- | --- | --- | --- | --- | --- | --- | --- | --- | --- | --- | --- |
|  |  | **25^th^ centile**  **59µg/g** | **50^th^ centile**  **83µg/g** | **75^th^ centile**  **121µg/g** | **25^th^ vs. 75^th^ centile difference** | **P _overall_^‡^** | **25^th^ centile**  **45µg/L** | **50^th^ centile**  **76µg/L** | **75^th^ centile**  **120µg/L** | **25^th^ vs. 75^th^**  **centile difference** | **P _overall_^‡^** |
| **Birthweight centile** | Estimate  (99% CIs) | 43.8  (41.4 to 46.3) | 45.0  (42.5 to 47.4) | 46.5  (44.1 to 48.9) | 2.69  (0.83 to 4.56) | 0.001 | 46.1  (43.6 to 48.6) | 45.7  (43.4 to 48.1) | 44.6  (42.1 to 47.1) | -0.91  (-3.05 to 1.16) | 0.020 |
| **SGA** | Percent  (99% CIs) | 11.6  (9.2 to 14.6) | 10.7  (8.3 to 13.5) | 9.8  (7.7 to 12.4) | -1.87  (-3.73 to -0.01) | 0.010 | 10.5  (8.2 to 13.3) | 10.5  (8.3 to 13.2) | 10.9  (8.4 to 13.9) | 0.10  (-2.11 to 2.30) | 0.731 |
| **Birthweight (grams)** | Estimate  (99% CIs) | 3309  (3267 to 3352) | 3328  (3286 to 3369) | 3350  (3310 to 3390) | 41  (13 to 69) | 0.001 | 3343  (3301 to 3385) | 3339  (3399 to 3380) | 3326  (3284 to 3368) | -8  (-39 to 22) | 0.042 |
| **Low birth weight** | Percent  (99% CIs) | 1.6  (0.9 to 2.9) | 1.5  (0.8 to 2.6) | 1.3  (0.7 to 2.3) | -0.31  (-0.82 to 0.19) | 0.226 | 1.4  (0.8 to 2.5) | 1.4  (0.8 to 2.5) | 1.5  (0.8 to 2.7) | 0.04  (-0.52 to 0.60) | 0.726 |
| **Head circumference (mm)** | Estimate  (99% CIs) | 344.6  (343.3 to 345.9) | 344.8  (343.5 to 346.2) | 345.3  (344.0 to 346.6) | 0.71  (-0.21 to 1.63) | 0.241 | 345.2  (343.9 to 346.5) | 345.1  (343.8 to 346.4) | 344.8  (343.4 to 346.2) | -0.31  (-1.28 to 0.67) | 0.440 |
| **Apgar 1 minute** | Estimate  (99% CIs) | 8.3  (8.2 to 8.5) | 8.3  (8.2 to 8.5) | 8.3  (8.2 to 8.4) | -0.02  (-0.12 to 0.07) | 0.690 | 8.3  (8.2 to 8.5) | 8.3  (8.2 to 8.5) | 8.3  (8.2 to 8.5) | 0.00  (-0.10 to 0.11) | 0.542 |
| **Apgar 5 minutes** | Estimate  (99% CIs) | 9.1  (9.0 to 9.2) | 9.1  (9.0 to 9.2) | 9.1  (9.0 to 9.1) | -0.01  (-0.06 to 0.04) | 0.533 | 9.1  (9.0 to 9.1) | 9.1  (9.0 to 9.1) | 9.1  (9.0 to 9.1) | -0.01  (-0.07 to 0.04) | 0.014 |
| **Ultrasound head circumference (mm)** | Estimate  (99% CIs) | 316.2  (314.4 to 318.1) | 316.5  (314.6 to 318.4) | 317.2  (315.4 to 319.0) | 1.0  (-0.4 to 2.3) | 0.084 | 316.7  (314.8 to 318.7) | 316.8  (315.1 to 318.7) | 316.7  (315.1 to 318.7) | 0.3  (-1.2 to 1.7) | 0.906 |
| **Ultrasound biparietal diameter (mm)** | Estimate  (99% CIs) | 8.62  (8.56 to 8.68) | 8.62  (8.56 to 8.68) | 8.63  (8.57 to 8.69) | 0.01  (-.04 to 0.06) | 0.911 | 8.62  (8.55 to 8.68) | 8.63  (8.56 to 8.69) | 8.64  (8.58 to 8.70) | 0.02  (-.03 to 0.07) | 0.268 |
| **Ultrasound femur length (mm)** | Estimate  (99% CIs) | 64.9  (64.5 to 65.4) | 64.9  (64.5 to 65.3) | 65.1  (64.7 to 65.5) | 0.2  (-0.2 to 0.5) | 0.478 | 65.0  (64.6 to 65.5) | 65.0  (64.6 to 65.5) | 65.0  (64.6 to 65.5) | 0.1  (-0.3 to 0.5) | 0.756 |
| **Ultrasound abdominal circumference (mm)** | Estimate  (99% CIs) | 297  (294 to 300) | 297  (294 to 300) | 298  (296 to 301) | 1  (-1 to 4) | 0.142 | 298  (295 to 301) | 298  (295 to 301) | 298  (295 to 300) | 0  (-2 to 3) | 0.013 |
| **Ultrasound estimated weight (grams)** | Estimate  (99% CIs) | 2269  (2220 to 2318) | 2275  (2226 to 2324) | 2297  (2251 to 2343) | 28.0  (-9.3 to 65.4) | 0.206 | 2291  (2242 to 2340) | 2292  (2246 to 2337) | 2285  (2237 to 2333) | 3.1  (-36.8 to 43.0) | 0.046 |
| **Preterm** | Percent  (99% CIs) | 4.7  (3.0 to 7.2) | 4.6  (2.9 to 7.1) | 4.3  (2.7 to 6.6) | -0.39  (-1.62 to 0.84) | 0.846 | 4.3  (2.7 to 6.8) | 4.5  (2.9 to 6.9) | 4.6  (2.9 to 7.2) | 0.53  (-0.81 to 1.87) | 0.226 |
| **Stillbirth** | Percent  (99% CIs) | 0.37  (0.07 to 1.90) | 0.36  (0.06 to 1.93) | 0.33  (0.07 to 1.59) | -0.44  (-0.37 to 0.28) | 0.834 | 0.32  (0.05 to 1.90) | 0.31  (0.06 to 1.71) | 0.31  (0.06 to 1.53) | 0.00  (-0.35 to 0.35) | 0.421 |
| **Congenital anomaly** | Percent  (99% CIs) | 6.5  (4.2 to 9.7) | 6.2  (4.2 to 9.3) | 5.5  (3.7 to 8.2) | -0.94  (-2.71 to 0.84) | 0.422 | 6.7  (4.4 to 10.0) | 6.4  (4.3 to 9.4) | 5.5  (3.6 to 8.4) | -0.87  (-2.89 to 1.15) | 0.170 |
| **Nervous system anomaly** | Percent  (99% CIs) | 1.8  (0.8 to 3.7) | 2.1  (1.0 to 4.1) | 1.9  (0.9 to 3.9) | 0.14  (-0.74 to 1.02) | 0.448 | 1.9  (0.9 to 3.9) | 2.0  (1.0 to 4.1) | 2.1  (1.0 to 4.5) | 0.23  (-0.93 to 1.40) | 0.120 |

*p-_overall_ is derived from comparing restricted cubic spline models with and without iodine status included. p<0·01 indicates a significant association between iodine and the outcome and the association may be linear or non-linear in nature.

† 'Average' participants were specified according to the largest categories or mean cohort values. Predicted estimates represent an average birth, not specified by Child's sex are relate to participants who are primiparous, white ethnic background, 'Employed and not materially deprived', did not smoke, drink or experience complications in pregnancy and have average BMI, age and gestation length.

‡ Note that p-values are unaffected by specifying 'average participants' and therefore relate to observed associations in the whole cohort.

Figure S1. A directed acyclic graph used to identify confounders and competing exposures in the association between maternal iodine concentration and birth or pregnancy outcomes


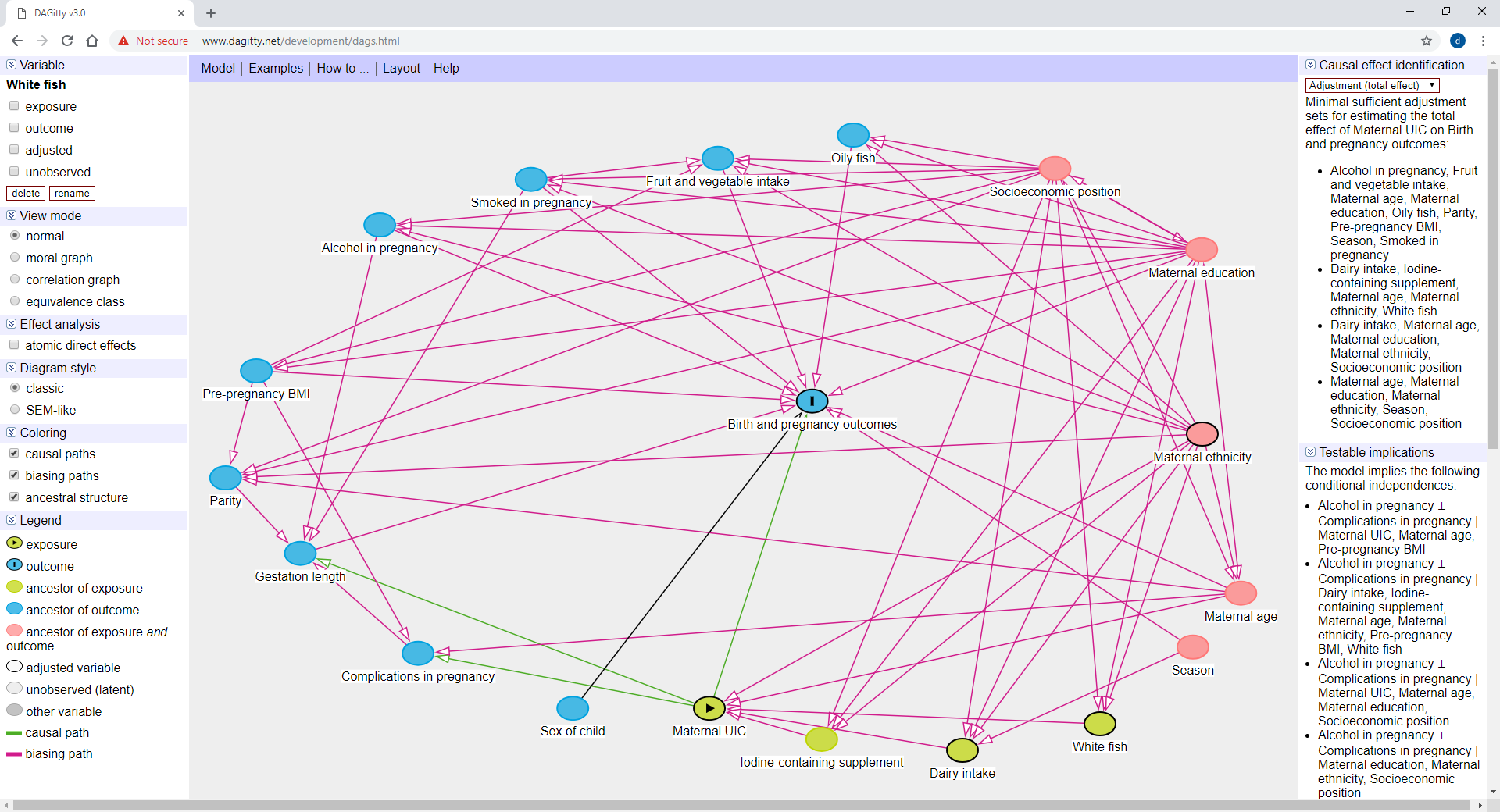


Constructed using DAGitty software.^38^

Stillbirths^1^ (n=37)

Figure S2: Flow chart of participant inclusions and exclusions.

^1^Still births were excluded from analyses except where ultrasound scan measurements or stillbirths were outcome. Any remaining variation in numbers analysed is due to small amounts of missing outcome or covariate data.

Ultrasound

measurements (n=1116)

Live births (n=6637)

Excluded:

- no linkage to child data (n= 47)

- twins and triplets (n=339)

- sample contamination or failed detection (n=6)

Mothers recruited into Born in Bradford cohort, 2007-2011 (n=12453)

Urine samples, 26-28 weeks gestation (n=7066 from 6644 mothers)

Analysable urine samples (n=6674 from 6355 mothers)

Pregnancies (n=13963)

Figure S3: Estimated APGAR score at 1 minute and percent with congenital anomalies, using imputed datasets for the full sample


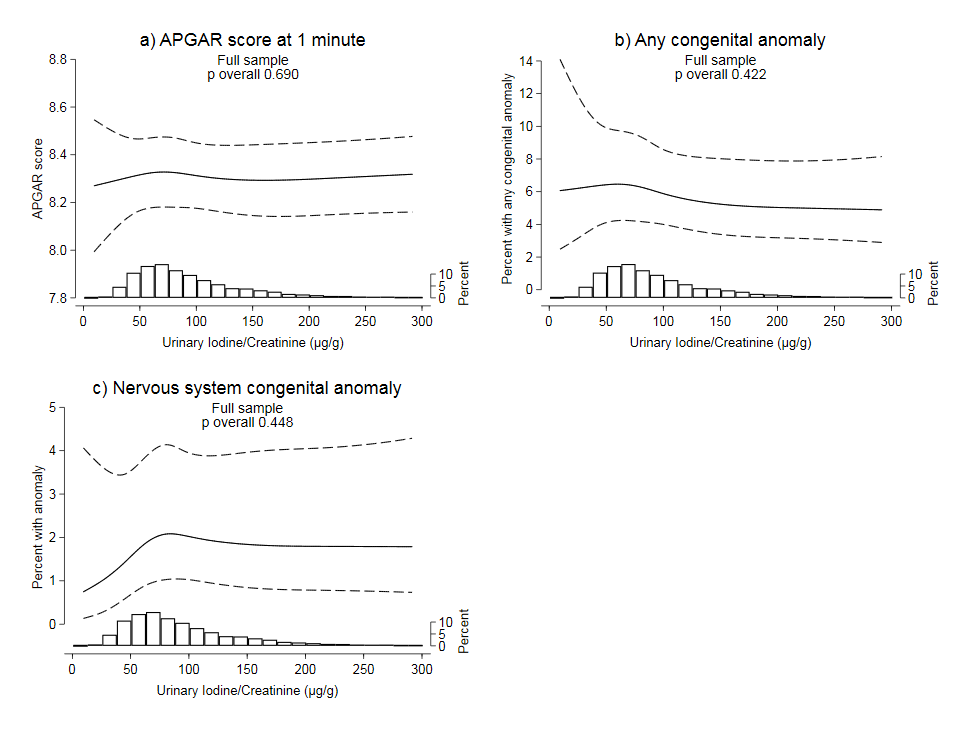


The spline position in these figures illustrates the predicted estimate for average participants (primiparous, white ethnic background, 'Employed and not materially deprived', did not smoke, drink or experience complications in pregnancy and have average BMI, age and gestation length).

Figure S4: Estimated biparietal diameter, femur length and abdominal circumference, from ultrasound scans at 34 weeks’ gestation, across the range of maternal I:Cr concentrations using imputed datasets for the full sample

The spline position in these figures illustrates the predicted estimate for typical participants (primiparous, white ethnic background, 'Employed and not materially deprived', did not smoke, drink or experience complications in pregnancy and have average BMI, age and gestation length).

Figure S5: Estimated birthweight centile in all participants, sensitivity analysis and subgroups


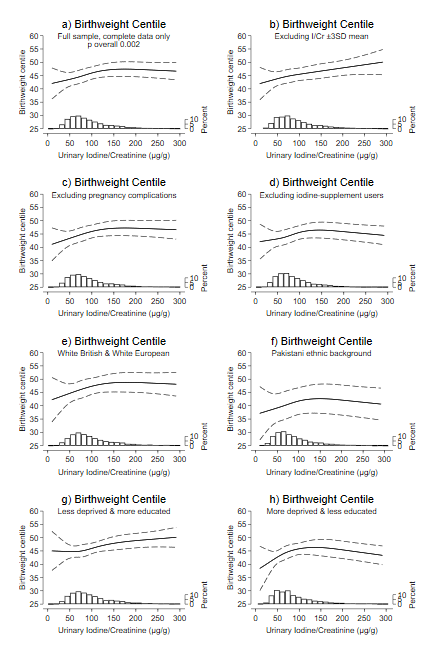
Subgroup interaction tests: ethnic group (p = 0.282); deprivation & education group (p = 0.008).

Figure S6: Estimated birthweight in grams in all participants, sensitivity analysis and subgroups


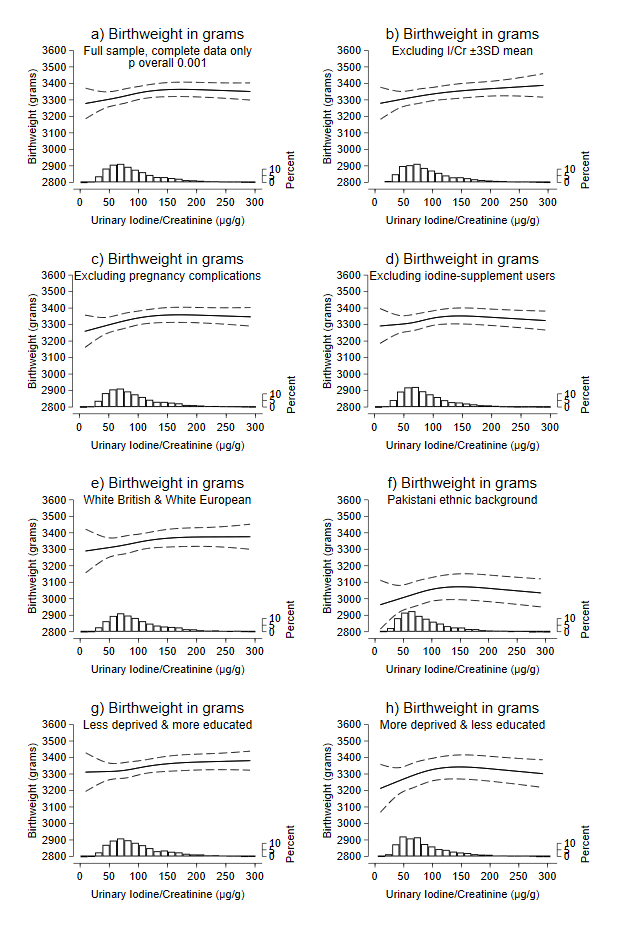


Subgroup interaction tests: ethnic group (p = 0.418); deprivation & education group (p = 0.081).

Figure S7: Probability of being small for gestational age in all participants, sensitivity analysis and subgroups


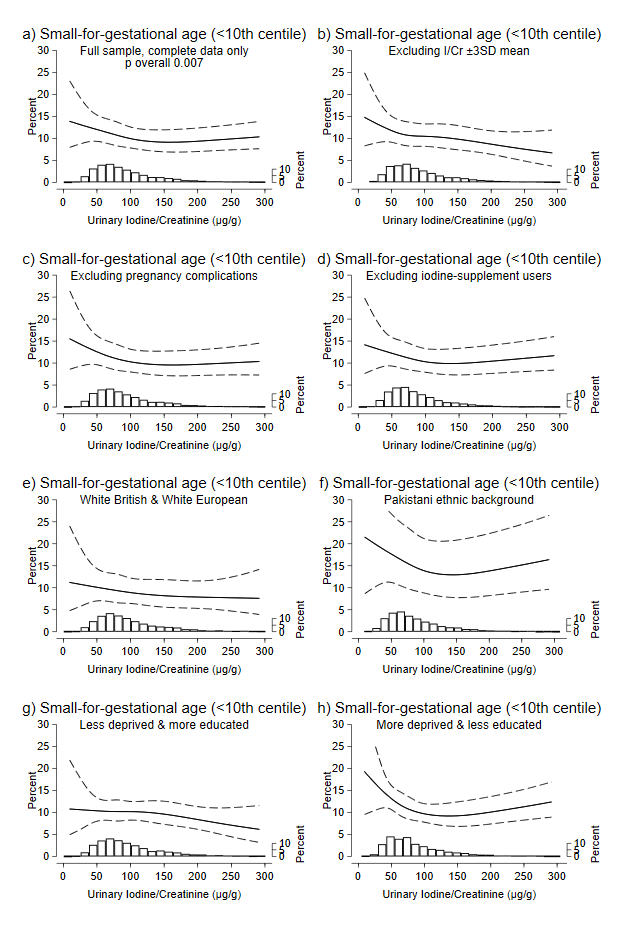


Subgroup interaction tests: ethnic group (p = 0.058); deprivation & education group (p = 0.026).

Figure S8: Probability of low birthweight in all participants, sensitivity analysis and subgroups


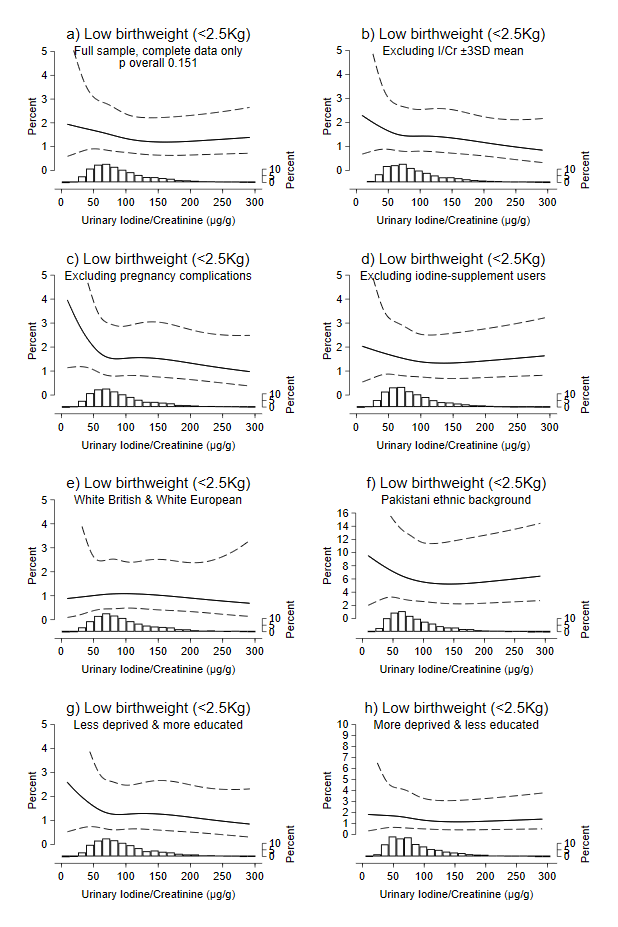


Subgroup interaction tests: ethnic group (p = 0.460); deprivation & education group (p = 0.288).

Figure S9: Apgar score at 5 minutes after birth in all participants, sensitivity analysis and subgroups


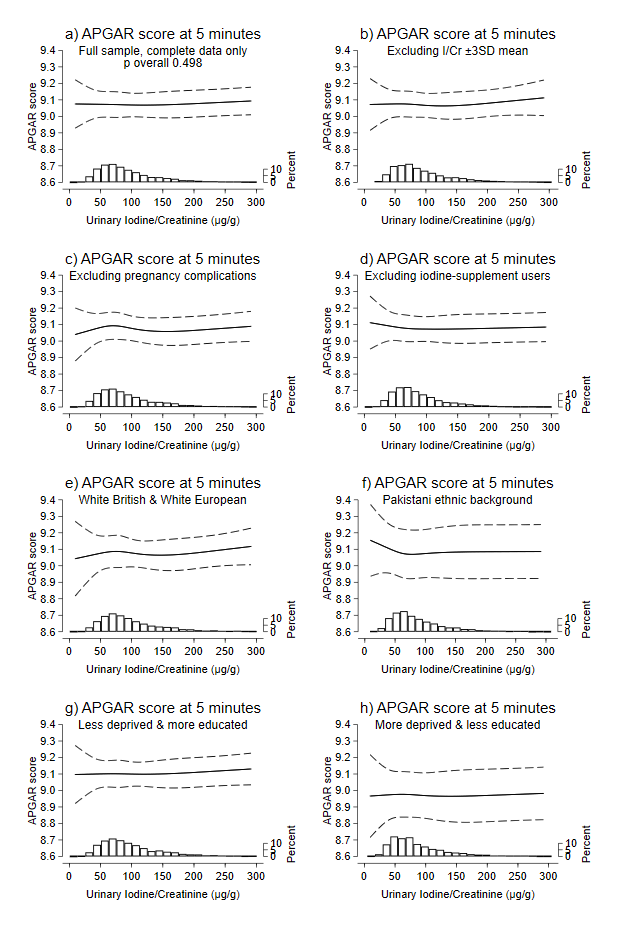


Subgroup interaction tests: ethnic group (p = 0.471); deprivation & education group (p = 0.971).

Figure S10: Estimated head circumference at birth in all participants, sensitivity analysis and subgroups


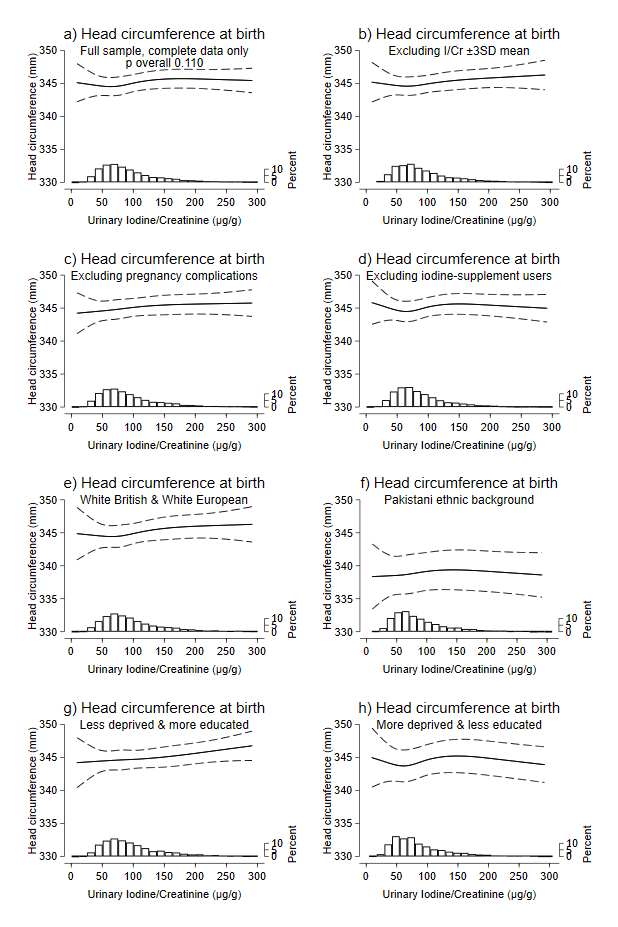


Subgroup interaction tests: ethnic group (p = 0.573); deprivation & education group (p = 0.007).

Figure S11: Estimated head circumference from 34 week ultrasound scan in all participants, sensitivity analysis and subgroups


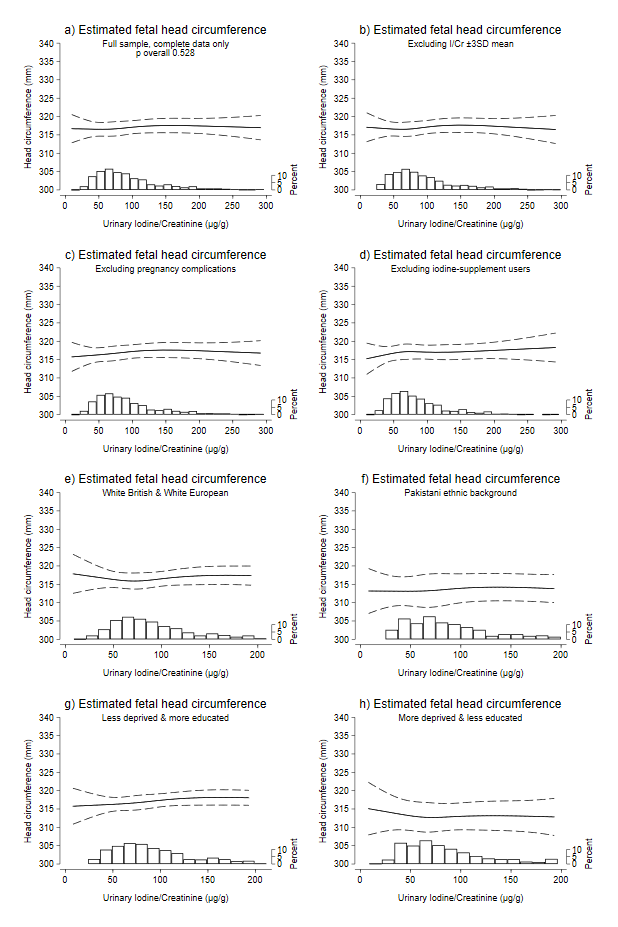


Subgroup interaction tests: ethnic group (p = 0.838); deprivation & education group (p = 0.366).

Figure S12: Estimated weight from 34 week ultrasound scan in all participants, sensitivity analysis and subgroups


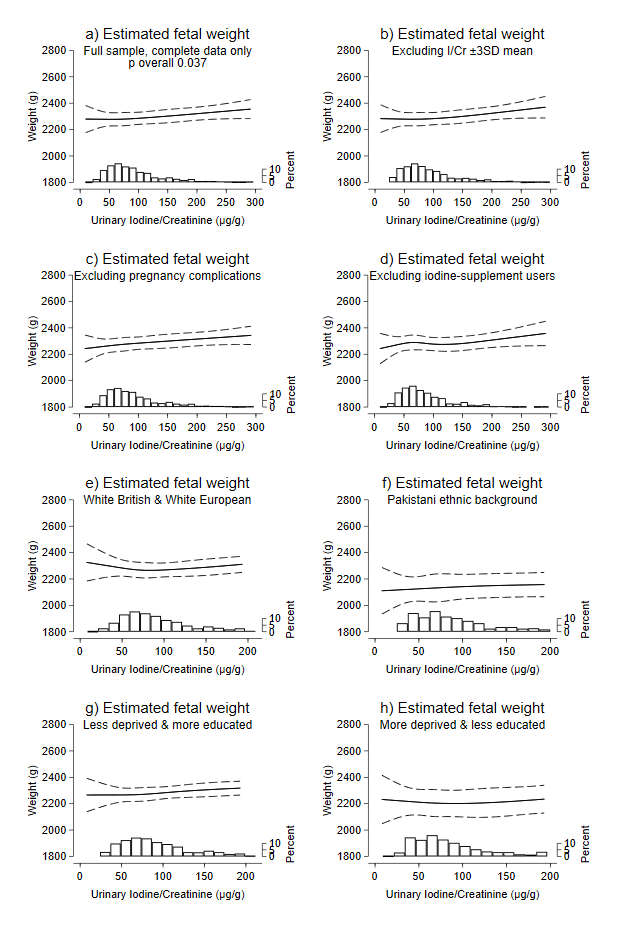


Subgroup interaction tests: ethnic group (p = 0.488); deprivation & education group (p = 0.800).

Figure S13: Probability of preterm birth in all participants, sensitivity analysis and subgroups


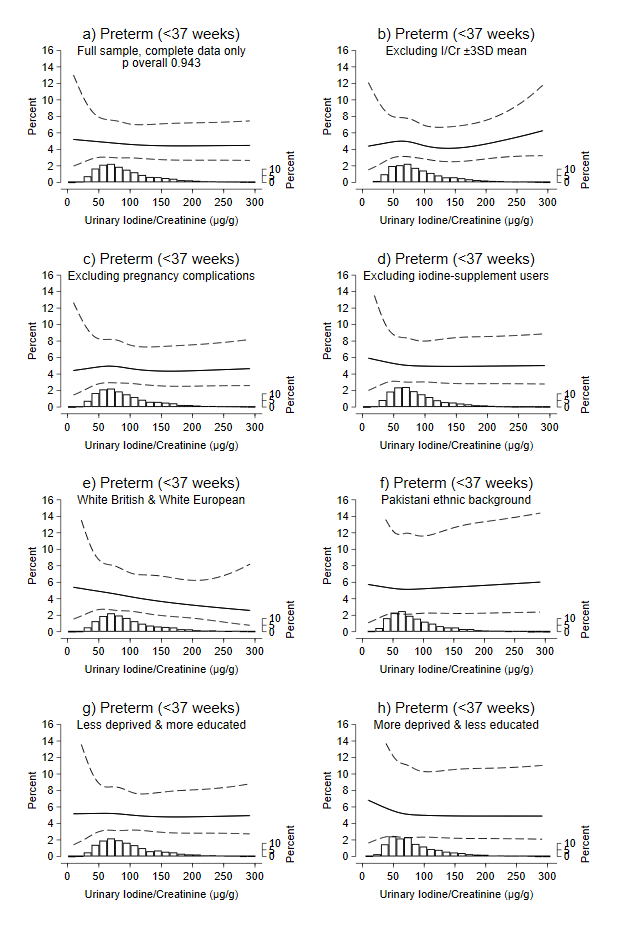


Subgroup interaction tests: ethnic group (p = 0.415); deprivation & education group (p = 0.954).

Figure S14: Probability of congenital anomalies in all participants, sensitivity analysis and subgroups


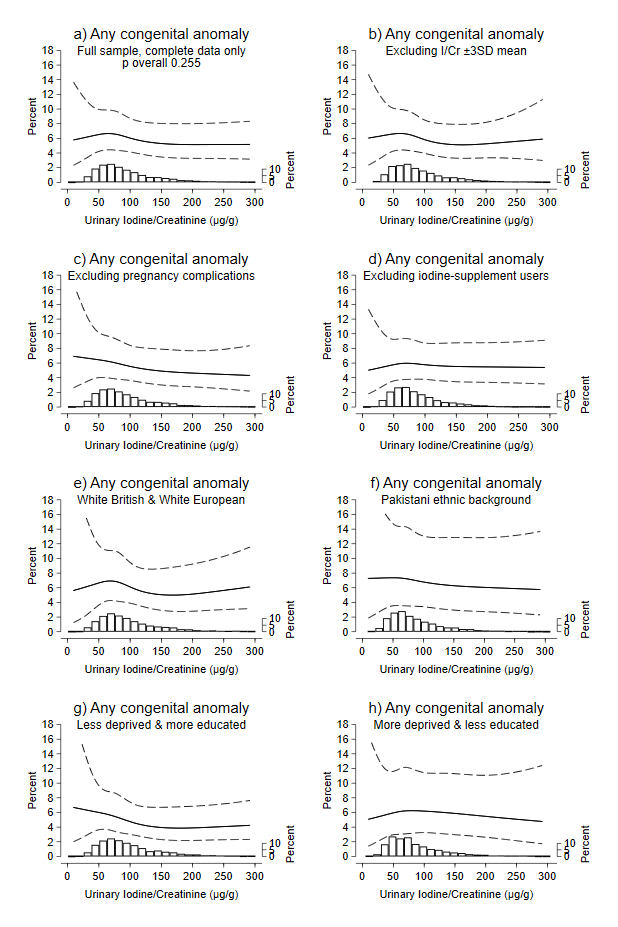


Subgroup interaction tests: ethnic group (p = 0.796); deprivation & education group (p = 0.131).
